# Does an ‘aspirin-a-day’ keep the doctor away?

**DOI:** 10.1038/sj.bjc.6690641

**Published:** 1999-09

**Authors:** H J Berkel

**Affiliations:** Feist-Weiller Cancer Center, Louisiana State University Medical Center, Shreveport, LA, USA


*British Journal of Cancer* (1999) **81**(1), 1–2
© 1999 Cancer Research Campaign
Article no. bjoc.1999.0641

Editorial

### Does an 'aspirin-a-day' keep the doctor away?

**HJ Berkel**

Feist-Weiller Cancer Center, Louisiana State University Medical Center, Shreveport, LA, USA

In this issue of the *British Journal of Cancer*, Collett et al (1999) report on an interesting, and until now mostly missing, piece of the jigsaw puzzle concerning the relationship between colorectal cancer risk and the use of non-steroidal anti-inflammatory drugs (NSAIDs).

Waddell, in 1983, was the first to report on the results of the treatment of four patients with multiple colorectal polyps with Sulindac, one of the drugs in the class of NSAIDs (Waddell and Loughrey, 1983). All his patients showed a remarkable regression and even disappearance of the polyps when given Sulindac. After the publication of this first case-series, another nine papers reporting on a total of 100 patients were published. It is quite remarkable that overall a 100% response rate was found in these 100 patients. That is to say, in all the patients the polyps in the large intestine either disappeared completely or greatly diminished in size and number. It also has to be noted that these initial patients treated with Sulindac were patients with polyposis related to hereditary conditions, such as Gardner's syndrome or familial polyposis coli, who had undergone a subtotal colectomy for their underlying disease. Therefore these patients are not really comparable to the average patient with a sporadic adenomatous polyp.

In addition to these early clinical results, a large number of animal-experimental studies was performed evaluating the effect of NSAIDs on colorectal cancer in a variety of animal models. These studies were done using different NSAIDs, under often different circumstances and with a variety of outcome and effect measures. Virtually all of the animal-experimental work confirms the results of the clinical case-series: administration of NSAIDs reduces the risk and the prevalence of indicators for colorectal carcinogenesis.

The next question was obviously to evaluate the effect – if any – of the use of these drugs on colorectal cancer risk in humans. Virtually all descriptive epidemiological studies (Berkel et al, 1996) have shown an inverse relationship between use of NSAIDs and subsequent colon cancer risk, suggesting a protective effect. Among the epidemiological studies were cohort studies, and case–control studies performed in different settings and among different populations. In addition, a reduced incidence of colorectal cancer was found in several populations who were often prescribed these drugs for other diseases, i.e. patients with rheumatoid disorders. All these pieces of the puzzle taken together make for an overall picture which is very suggestive of a cause–effect relationship. Several pieces of the puzzle were still missing, however, and questions remained in particular with regard to dose–response and duration of use required to have a protective effect; in particular, since in the only reported intervention study, the Physicians Health Study, no beneficial effect of low-dose aspirin (325 mg on alternate days) on colorectal cancer risk was observed after 5 years of follow-up (Gann et al, 1993). In the Nurses Health Study a significant trend in protective effect was found with longer duration of use among women who took ≥2 aspirins (Giovannucci et al, 1995). The largest risk reduction was found in women who used aspirin consistently for > 20 years (relative risk = 0.56).

The question of induction and latent period ('how long does it take before an effect is evident?') was eloquently addressed in the study by Collet, reported in this issue of the journal (Collet et al, 1999). In a non-concurrent cohort linkage study, linking the population-based cancer registry with the database of the province-wide drug prescription plan in the province of Saskatchewan, Canada, the investigators were able to show that it took ≥10 years for a protective effect of NSAID use to become apparent. Although this result seems to be consistent with the earlier results from the Nurses Health Study, there are some questions about the reliability of their conclusions: because of the fact that the investigators used the Drug Prescription database, there is a distinct possibility for mis-classification bias. On the one hand there is an obvious underestimate of exposure: no data are available about so-called 'over the counter' drug use, and since aspirin is an over the counter drug and is/has been widely available without prescription for a large mixed group of disorders, a differential trend in use over time could seriously impact the results. On the other hand, being given a prescription does not necessarily mean that the drugs are indeed used. In addition, since the investigators choose not to evaluate – for a variety of good reasons – the effects of individual NSAIDs, but rather use a composite measure of overall use, it is not clear which of the NSAIDs – if any – is preferable.

Despite the potential pitfalls of this study, it contributes significantly to the ever growing body of research which has suggested that NSAIDs have a protective effect on colorectal cancer development in humans. At least three patho-physiological mechanisms have been suggested to explain this beneficial effect. Firstly, it appears that certain prostaglandins, in particular prostaglandin $E_2$, can inhibit cellular immune responses which are important in the host defense against malignant cells. NSAIDs, through their inhibitory effect on the cyclo-oxygenase (COX) enzymes, block the production of prostaglandin $E_2$ and thus may inhibit prostaglandin-dependent immunosuppression, allowing augmentation of the anti-tumour aspects of the cellular immune response. Secondly, through its inhibitory effects on the two COX enzymes, COX-1 and COX-2, the arachidonic acid metabolism is influenced. Marnett (1992) concluded that there was overwhelming support for the notion that influences on arachidonic acid metabolism contribute to the carcinogenetic process in humans and that it is therefore possible to modulate carcinogenesis through, for example, the use of COX-inhibitors. Finally, recently it has been suggested that NSAIDs may exert their protective effect through an effect on apoptosis. Apoptosis, or programmed cell death, and the relation between cell division and cell death are believed to be crucial in tumorigenesis. Defective regulation of apoptosis can promote the tumour development. Several metabolites of NSAIDs have been shown to inhibit cell cycle progression without significantly reducing prostaglandin $E_2$ levels, which may indicate an effect on apoptosis. It seems unlikely that any of these mechanisms in and by themselves are responsible for all of the potentially beneficial effects of NSAID use, rather it can be expected that the patho-physiological mechanisms underlying the effects of NSAIDs are multifactorial.

One issue which the study by Collett et al (1999) did not, and could not, address is the concern about side-effects of these drugs. Aspirin and the other NSAIDs are well known for their side-effects, in particular on the upper gastrointestinal tact. The most severe of these complications is bleeding peptic ulcers. It is not acceptable to advocate a (chemo-)preventive strategy to the population-at-large when a high risk for major side-effects exists. In that regard the development of more specific COX-2 inhibitors is of great interest. These drugs, the first of which has recently been approved by the Food and Drug Administration in the USA for use in patients with rheumatoid arthritis, are suggested to have many fewer gastrointestinal side-effects, while at the same time not having lost their therapeutic/preventative benefits.

The crucial question now becomes, as the title of this editorial indicates, does the use of NSAIDs indeed prevent colorectal cancer, and should our patients be advised to use this drug in an effort to prevent colorectal cancer? The proof-of-the-pudding, as in many instances before, is in the eating. An impressive body of evidence has been assembled suggesting a preventative effect of NSAIDs on colorectal cancer risk, but the ultimate proof has not yet been provided. We now need to proceed to the next logical step in the sequence of research: the intervention study. The story of β-carotene which showed great promise as a chemo-preventive agent for lung cancer based on results of observational epidemiological research, but was found not only not to decrease, but potentially even to increase lung cancer risk, should remind all of us that without positive results from solid randomized intervention trials, it remains premature to already advise our patients to take ‘an aspirin a day’ to prevent colorectal cancer. Some of these trials are underway already and it is prudent to await the results of these studies before advocating the use of NSAIDs for the prevention of colorectal cancer.

### REFERENCES

Berkel J, Holcombe RF, Middlebrooks M and Kannan K (1996) Non-steroidal anti-inflammatory drugs and colorectal cancer. *Epidemiol Rev* **18**: 205–217

Collet J-P, Sharpe C, Belzile E, Boivin J-F, Hanley J and Abenhaim L (1999) Colorectal cancer prevention by nonsteroidal antiinflammatory drugs: effects of dosage and timing. *Br J Cancer* **80**: 00–00

Gann PH, Manson JE, Glynn RJ, Buring JE and Hennekens CH (1993) Low-dose aspirin and incidence of colorectal tumors in a randomized trial. *J Natl Cancer Inst* **85**: 1220–1224

Giovannuci E, Egan KM, Hunter DJ, Stampfer MJ, Colditz GA, Willett WC and Speizer FE (1995) Aspirin and the risk of colorectal cancer in women. *N Engl J Med* **333**: 609–614

Marnett LJ (1992) Aspirin and the potential role of prostaglandins in colon cancer. *Cancer Res* **52**: 5575–5589

Sheng H, Shao J, Kirkland SC, Isakson P, Coffey RJ et al (1997) Inhibition of human colon cancer cell growth by selective inhibition of cyclooxygenase-2. *J Clin Invest* **99**: 2254–2259

Waddell WR and Loughrey RW (1983) Sulindac for polyposis of the colon. *J Surg Oncol* **24**: 83–87

*© 1999 Cancer Research Campaign*

